# Culturally tailored postsecondary nutrition and health education curricula for indigenous populations

**DOI:** 10.3402/ijch.v72i0.21144

**Published:** 2013-08-05

**Authors:** Sarah McConnell

**Affiliations:** University of Alaska, Anchorage, Alaska, USA

**Keywords:** cultural tailoring, postsecondary education, nutrition, indigenous, health, health literacy

## Abstract

**Background:**

In preparation for the initial offering of the University of Alaska Fairbanks (UAF), Interior–Aleutians Campus Rural Nutrition Services (RNS) program, a literature review was conducted to establish the need for the proposed program and to substantiate the methodology for delivering integrated, culturally tailored postsecondary education and extension to Alaska Natives and rural Alaskans. There was a striking absence of peer-reviewed journal articles describing culturally tailored postsecondary health curricula for indigenous populations.

**Objective:**

To complete and discuss a current (November 2012) literature review for culturally tailored postsecondary health curricula designed and delivered for indigenous populations.

**Methods/Design:**

The author conducted an expanded online search that employed multiple configurations of key terms using Google and Google Scholar, as well as pertinent sources. The author located archived reports in person and contacted authors by email.

**Results:**

The expanded search produced a modest amount of additional literature for review. A disappointing number of publications describing or evaluating culturally tailored postsecondary health curricula in mainstream institutions are available. Related resources on culturally tailored extension and resources for the development and delivery of culturally tailored nutrition and health curricula were identified.

**Conclusions:**

The present results demonstrate a significant absence of literature on the topic, which may or may not indicate the absence of sufficient culturally tailored postsecondary health curricula for indigenous populations. There are indications that culturally tailored postsecondary health curricula for indigenous populations have the potential to effectively address certain issues of health literacy and health disparities.

In preparation for the initial 2008 offering of the University of Alaska Fairbanks (UAF), Interior–Aleutians Campus Rural Nutrition Services (RNS) program, a literature review was conducted to establish the need for the proposed program and to substantiate the methodology for delivering integrated, culturally tailored postsecondary education and extension to Alaska Natives and rural Alaskans. A majority of the supporting postsecondary health curriculum resources available were unpublished reports and “personal communications” or oral histories from Alaska Native elders, regional Alaska Native health system leaders, University of Alaska (UA) faculty and adjuncts, past and current Alaska Native UA students, and community members living in remote rural Alaskan villages, many of them visited by chronic health problems such as diabetes or cardiovascular disease. There was a striking absence of peer-reviewed journal articles describing culturally tailored postsecondary health curricula for indigenous populations. In fact, only one pre-publication peer-reviewed article, generously shared by the article's author, was found to describe culturally tailored American Indian/Alaska Native (AI/AN) postsecondary nutrition education that included priority elements of cultural tailoring such as Native elders as teachers/mentors and a focus on traditional foods for health (1). The United States Department of Agriculture (USDA), National Institute of Food and Agriculture, National Research Initiative (award #2008–55215–18781) funded the UAF project, which has been serving Alaska Native and rural Alaskan students since 2008. Data collected in April 2012 showed that 94% of students self-identified as AI/AN and demonstrated a 91% completion rate in 499 academic credit hours, as well as a 77% success rate by students that attempted to complete the full Occupational Endorsement (OE) credential. A current (November 2012) literature review has been completed and discussion follows.

Indigenous populations experience a disproportionate occurrence of chronic diseases of nutrition and lifestyle (2). These diseases are frequently associated with the change from traditional diets to standard American or Western diets that, along with health promoting foods, include added sugars, increased refined carbohydrates, saturated fats, and an array of additives alien to traditional indigenous diets. Mainstream nutrition and health curricula reviewed during RNS development typically did not fully address the complex cultural, social, physical, and spiritual context of indigenous diets, health promotion, health problems, and healing. Mainstream postsecondary nutrition and health teaching methodologies tended to employ teaching and learning styles that lack resonance for many indigenous learners. Common indigenous values, ways of knowing, and methods that promote learning have been difficult to find in mainstream postsecondary education. These values include, but are not limited to, spirituality; respect for elders; personal relationships with land, waterways, animals, and plants; and sharing. Learning methods include, but are not limited to, storytelling, valued personal observation and experience, visual design, singing, and movement. Mainstream postsecondary nutrition and health education does not typically provide space or support for indigenous students to address classroom issues of cultural dissonance, decolonization, or the lasting impacts of historical trauma that may pose barriers to personal health, learning, and ultimately, health service delivery.

There is a need to build a local, credentialed workforce that understands the indigenous cultural context, food system, ecology, and language in order to support health literacy, while increasing retention of health knowledge and skills in remote, rural, and/or underserved indigenous communities. Culturally tailored postsecondary nutrition and health curricula leading to academic credentials are a promising pathway to building capacity to respond within the ecology and cultural context of a community. Building local capacity has been demonstrated to be an effective intervention in circumpolar indigenous health, as evidenced by the Community Health Aide Program (CHAP) initiated in the 1950s as one of the components of Alaska's response to tuberculosis (3,4).

## Review methods

An extensive online search employing multiple configurations of key terms including, but not limited to, “culturally tailored,” “postsecondary,” “university,” “indigenous,” “curriculum,” “nutrition,” “health,” “academic credential,” “culturally relevant,” and “culturally meaningful” was conducted using Google and Google Scholar. There was also a specific online search of pertinent sources including, but not limited to, PubMed, the *International Journal of Circumpolar Health*, and the *Journal of the Society of Nutrition Education and Behavior*. Additional review was accomplished by examining publication references and then searching for selected references that appeared as though they might yield relevant information. Furthermore, in person, the author located archived reports on the UAF Rural Human Services Program (RHSP).

The specific target of this review is “postsecondary culturally tailored nutrition (and health) curricula for indigenous populations and leading to an academic credential.” The review will primarily address mainstream university nutrition and health curricula, including behavioral and environmental health, and, in particular, identify curricula leading to a credential that is part of an academic pathway to advanced degree/s. Examples of culturally tailored extension, resources reviewing cultural competence in postsecondary curricula for indigenous populations, and a few resources to guide development of culturally tailored curriculum will also receive brief mention. It is readily acknowledged that additional culturally tailored postsecondary health curricula for indigenous populations exist but are not published or were not found for this review. Indeed, a major purpose of this review is to stimulate publication and information exchange regarding this topic.

This literature review casts a wider net than the pre-2008 review in an effort to identify more postsecondary academic credential curricula for community nutrition and health promotion that is culturally tailored to indigenous populations. In order to locate resources, the parameters widened were geographic and disciplinary. The search expanded from AI/AN regions to include indigenous people of circumpolar regions, as well as Native Hawai'ian, Maori, and Australian Aboriginal peoples; it expanded from nutrition science education to include community health delivery, environmental health, and behavioral health. The search focused on “mainstream” postsecondary institutions and did not include tribal or First Nations institutions because it is assumed that they inherently educate from an indigenous perspective. The goals of this literature review are to provide a critical analysis of identified works as they contribute to the establishment of culturally tailored postsecondary nutrition and health education methodologies as effective academic and workforce preparation tools; provide a foundation for further contributions of peer-reviewed literature; and, ultimately, advance integrated, culturally tailored postsecondary education and extension as an essential, effective, and accepted method to build indigenous student engagement in postsecondary education, while addressing health disparities and health literacy among indigenous populations in circumpolar regions and beyond.

No other literature reviews on this specifically defined subject were found. An apparently thorough multinational review of cultural competence and cultural safety in indigenous public health curriculum related to the Competencies for Indigenous Public Health Evaluation and Research (CIPHER) project looks at curricula in the United States, Canada, Australia, and New Zealand and is worthy of note (2). A somewhat related literature review addressing indigenous health workforce inequities and focusing on “best” practice for recruitment into tertiary health programs was studied (5), but it did not yield detail specific to the target of this review. Also available is a 2003 United States Health Resources and Services literature review on the Community Health Aide Program (CHAP) (4). Though not a fully culturally tailored postsecondary curriculum as defined in this review, the CHAP training that emerged in the 1950s represents important contributions (3,4) and did provide much of the valuable initial inspiration for the Rural Human Services program that is of substantial interest in the current literature review.

## Findings

As a result of this review, use of the term “culturally tailored” in this article will refer to planned, conscious development and delivery of academic coursework that includes many or all of the following elements: traditional indigenous lifeways such as diet, food acquisition, family practices, healing, physical activity, indigenous spirituality, and/or resource management; inclusion and development of indigenous language materials; utilizing indigenous educators or persons experienced with the culture in substantive roles; including indigenous elders as teachers or mentors; respect for indigenous protocols; seeking approval and involvement of the indigenous community; showing respect and applying indigenous values, beliefs, and behaviors; and indigenous ways of knowing and teaching as central to the educational process. Cultural tailoring may well exceed the preceding possibilities. Cultural tailoring promotes the foundation “… assumption that there are different but equitable sources of knowledge and ways of knowing” (6, p. 89). For the purpose of this review, cultural tailoring indicates full integration of the indigenous worldview and knowledge systems, including the indigenous perspective of education healing and wholeness, spirituality and learning life skills—useful skills (1). It is to be distinguished from mainstream curriculum that may add on visible tangible levels of artifacts and objects without acknowledging the importance of indigenous knowledge or learning processes. Further, we find in the culturally tailored curricula a sense of commitment to address such issues as cultural survival, social justice, decolonization, historic trauma, and cultural dissonance as essential parts of the educational process.

In addition to the RNS program, that has not been published, four culturally tailored postsecondary health curricula were identified. Culturally tailored curricula leading to an academic health-related credential included the Rural Human Services program, first delivered at UAF in 1991 (7) and the Woodlands Wisdom (Nutrition) program at the University of Minnesota (1) that were seminal in the creation of the RNS program. Much of the RNS success is owed to the pioneering efforts of these academic programs. With the expanded search, two more distinctive programs were identified. A longstanding Early Childhood Care and Development program of the University of Victoria in British Columbia's School of Child and Youth Care (SCYC) began in 1991 in collaboration with the Meadow Lake Tribal Council of northern Saskatchewan; it is also referred to in the literature as the “Generative Curriculum” (6,8,9). Documentation was identified in the literature describing a fourth program, the Indigenous Environmental Studies (IES) program at Trent University in Canada (10).

The aforementioned authors provide an outstanding description of the cultural tailoring evidenced in their programs, and they include profound explanations of critical educational issues of decolonization, indigenous values, cultural survival, racism, and historic trauma, to name a few. They clearly address the essential importance and application of indigenous epistemologies and pedagogies. The programs evidence a high level of consistency across disciplines as well as geographical regions. The confines of this literature review do not do justice to the aggregate wisdom and experience of these authors and the respective indigenous participants’ contributions to their scholarly work. The reader is strongly encouraged to explore these works in their own rich detail.

Although culturally tailored stand-alone courses can certainly advance indigenous education, this review prioritized postsecondary curriculum that result in a specific academic credential and that is part of an academic pathway to advanced degrees. This is important in order to support students that complete culturally tailored programs in attaining the requirements to successfully compete for jobs in their community or region, or who advance to leadership positions that may require an education qualification. Ball does an excellent job of articulating the importance of this dynamic when she states that “First Nations are all too familiar with dead end training programs, which are typically short-term, skill-based programs that do not lead to transferable academic credits or recognized credentials” (6, p. 97–98). Bringing integrated cultural wisdom and discipline expertise to the workforce in indigenous communities is a promising avenue to address health disparities and health literacy issues. The RHS program represents the first step in an exemplary articulated academic pathway that is also coordinated with the career pathway recognized state-wide in behavioral health. Completion of the RHS curriculum results in a UA certificate that articulates to the associate's degree in human services, which leads to the bachelor's degree in social work or psychology, and on to the master's degree in social work (7,11). The unpublished RNS program has made strides toward modeling the RHS program articulation but with greater variety available to the student. Students can use their RNS OE to advance to a certificate in tribal management, and to an associate's degree in tribal management, educator paraprofessional, interdisciplinary studies, or science. RNS OE graduates pursuing a nutrition education pathway can articulate to the associate's degree of science or directly to the bachelor's of science degree in dietetics with fully portable credits from their OE. The Woodlands Wisdom Program results in an associate's degree with articulation to the bachelor's of science degree in dietetics (1). The SCYC program leads to a certificate in early childhood education and then a diploma in child and youth care, which may also be used to proceed to a higher degree in the same discipline. The SCYC program has clearly succeeded in aligning their academic program with workforce needs and opportunities in indigenous communities (6). The Trent University Indigenous Environmental Studies (IES) program leads to two-year diploma (10).

**Table:** 

Program	Cohorts	Indigenous Instructors	Elders	Food	Storytelling (personal experience or observation)	Relevant Indigenous Materials	Community as Teacher: Share & Learn	Learning styles— Experiential, Interactive, & Relational
RNS	Yes	Mix	Yes	Yes	Yes	Yes	Yes	Yes
RHS	Yes	Mix	Yes	Yes	Yes	Yes	Some	Yes
WWP		As available	Yes	Yes	Yes	Yes		Yes
SCYC	Yes		Yes		Yes	Yes	Yes	Yes
IES		Yes	Yes		Yes	Yes		Yes

RNS=Rural Nutrition Services, University of Alaska Fairbanks; RHS=Rural Human Services, University of Alaska Fairbanks (7,11,12); WWP=Woodlands Wisdom Program, University of Minnesota (1); SCYC=School of Child and Youth Care, University of Victoria, British Columbia (6,8,9); IES=Environmental Studies, Trent University (10).

The following chart attempts to establish some of the commonalities amongst the programs, as specified in the articles reviewed:

It is of particular note that two of the programs, RHS (7,11,12) and SCYC (6,8,9) have been established for about 20 years and offer significant potential to substantiate the need and efficacy of culturally tailored postsecondary curriculum in addressing the behavioral health and childcare needs of indigenous communities. These programs have made substantial impacts on indigenous representation in their respective workforces. Related support comes from the comprehensive multinational public health training review provided by Baba (2) in her focus on cultural competence and cultural safety, firmly advancing the premise that health disparities mitigation and indigenous health curriculum are exquisitely linked.

Clearly, there is a gap in the existing identifiable literature describing, evaluating, and advancing the knowledge base of culturally tailored postsecondary health curriculum for indigenous populations with the ultimate goal of training and credentialing health and human service providers to serve indigenous people. Seeking additional supporting resources, a few peripheral articles of note have been identified, and it is most probable that more exist. Worth mentioning in the area of culturally tailored extension for indigenous populations are nutrition and physical activity efforts led by Afele-Fa'amuli (13) in American Samoa and diabetes nutrition activities reported by Abbott (14) in Australia. In Afele-Fa'amuli's article, she presents concrete examples of cultural tailoring of health promotion extension and describes the effectiveness of cultural tailoring for an indigenous population (13). Abbott provides a research brief that is helpful in describing additional elements of cultural tailoring for an indigenous population and contributes useful lessons learned (14).

For readers with an interest in developing and delivering a culturally tailored postsecondary health curriculum, it seems worthwhile to include references to a few foundation works that provide reliable guidance. The collaborative work of Ray Barnhardt and the late Angayuqaq Oscar Kawagley is seminal in much of the culturally tailored postsecondary health curriculum found in the literature and collected in their more recent publications (15,16). The autonomous work of Kawagley (17) provides a broad foundation that includes detailed examples of the interconnectedness of culture, education, and wellness. In addition to sharing their wisdom, Barnhardt and Kawagley, importantly, provide a vehicle for indigenous elders, educators, and students to communicate directly to the reader, describing essential components of culture in meaningful, effective education (15). The Alaska Native Knowledge Network provides great detail in their *Cultural Standards and Guidelines* series (eight booklets) that includes *Standards for Culturally Responsive Schools* and *Guidelines for Respecting Cultural Knowledge*, *Strengthening Indigenous Languages*, and *Nurturing Culturally Healthy Youth* and is available in print, on CD, and online (18). It is of note that the booklets have all been approved by the Assembly of Alaska Native Educators and are endorsed by significant organizations such as the Alaska Federation of Natives. Cajete further advances the discussion specific to traditional knowledge as well as indigenous science and health perspectives in his book focusing on Native science and natural laws (19). He addresses the ecology of Native healing as well as discussion of indigenous nutrition. These authors contribute significantly to the understanding of “worldview” as well as indigenous epistemology and pedagogy, providing a bridge to effective cultural tailoring in mainstream postsecondary classrooms (15–19).

Additional writings potentially helpful to curriculum designers abound in the bycatch of the specific search for this review. An effort will be made to highlight a few, including those that appear in a volume of the *Canadian Journal of Education* (20,21). The use of cohorts is a common thread in culturally tailored academic credentialing programs, as described to some extent in the program descriptions noted here. Van der Wey expands on dynamics of coalition building as she describes challenges and benefits as well as establishment of a “safer discursive arena” (20). Though more focused on K–12 education, Saunders and Hill provide a curriculum model tailored for First Nations students and discuss steps to “… achieve reparative or equitous outcomes through the creation of in-class coalitions …” (21, p. 1015). Other guiding work comes from postsecondary health curriculum generated in the United States Tribal College network such as the unpublished Seven Generations Project at Southwest Indian Polytechnic Institute and a promising publication from the Quality Education for Minorities (QEM) Network: *Scholarly Guideposts for TCUP (Tribal Colleges and Universities Program) Faculty* (22). This publication contains guidance that includes strategies for integrating indigenous cultures in culturally tailored college science courses, mathematics courses, and an emphasis on culturally relevant evaluation.

The summary of literature that is specific to culturally tailored postsecondary nutrition and health curriculum, combined with related literature describing development and delivery of culturally tailored education, as well as literature describing some specific components of culturally tailored education all contribute to the acknowledgment of culturally tailored curriculum methodologies as effective academic and workforce preparation tools for indigenous populations. The articles cited provide a foundation for further identification of literature and contributions of peer-reviewed literature because it is evident that there is a shortage of specific nutrition and health curriculum descriptions as well as indigenous evaluation of such curriculum. Available literature does advance integrated culturally tailored postsecondary education and extension as an important, effective, and vital method to address health disparities among indigenous populations in the circumpolar regions and beyond.

## Discussion

This review has been conducted with enormous respect for the culturally tailored postsecondary health curricula studied, the efforts of the academic programs described and their students, and all the authors’ efforts in the field of indigenous education. My apologies to those representing culturally tailored mainstream postsecondary curricula that may have been unintentionally overlooked in this review and for any unintentional misinterpretations of the authors’ work.

Why is there such a limited availability of literature addressing culturally tailored postsecondary health curricula that lead to an academic credential in mainstream education? A wide range of possible explanations exists and exceeds what can be noted here. Some professionals delivering postsecondary nutrition and health education that is culturally tailored for indigenous populations are in bipartite appointments that do not include efforts for program documentation through scholarly writing and publication in peer-reviewed materials. The predominance of indigenous oral tradition may indirectly discourage written documentation of cultural perspectives that are part of the cultural tailoring. Some of the most successful culturally tailored programs, in terms of course and credential completion, and their effectiveness in reaching rural and remote indigenous communities with health outreach information, may be underfunded and understaffed—leaving no time for academic writing. Authentic reporting on program success absolutely requires genuine collaborative writing with participant students and indigenous community members who observe the outcomes. This collaborative approach is exceptionally time-consuming yet worth the investment because of the cultural depth, richness, and insightfulness that results as indigenous participants make observations, connections, interpretations, and conclusions an outsider may not even imagine. Whatever the barriers, it is hoped that this review will in some small measure stimulate increased efforts to develop, deliver, and describe culturally tailored postsecondary curriculum for indigenous populations, leading to academic credentials in the broad range of health and social programs.

## Conclusion

This literature review revealed a significant lack of publications specific to culturally tailored nutrition and health postsecondary curriculum for indigenous populations. Two notable effective, long-term academic programs were identified in related disciplines. Development and delivery of culturally tailored health and nutrition education has promising potential for successful education and workforce progress toward addressing health disparities and health literacy issues among indigenous populations and should be fully explored.
